# Compliant vascular models 3D printed with the Stratasys J750: a direct characterization of model distensibility using intravascular ultrasound

**DOI:** 10.1186/s41205-021-00114-8

**Published:** 2021-09-03

**Authors:** Adam J. Sparks, Cody M. Smith, Ariana B. Allman, Jillian L. Senko, Karen M. Meess, Richard W. Ducharme, Michael E. Springer, Muhammad Waqas, Adnan H. Siddiqui

**Affiliations:** 1 The Jacobs Institute, Buffalo, New York USA; 2 Department of Neurosurgery, University at Buffalo, State University of New York, 100 High Street, Suite B4, Buffalo, NY 14203 USA; 3 Canon Stroke and Vascular Research Center, University at Buffalo, State University of New York, Buffalo, New York USA

**Keywords:** 3D-Printing, Vasculature, Compliance, Intravascular Ultrasound, Distensibility

## Abstract

**Purpose:**

The purpose of this study is to evaluate biomechanical accuracy of 3D printed anatomical vessels using a material jetting printer (J750, Stratasys, Rehovot, Israel) by measuring distensibility via intravascular ultrasound.

**Materials and methods:**

The test samples are 3D printed tubes to simulate arterial vessels (aorta, carotid artery, and coronary artery). Each vessel type is defined by design geometry of the vessel inner diameter and wall thickness. Vessel inner diameters are aorta = 30mm, carotid = 7mm, and coronary = 3mm. Vessel wall thickness are aorta = 3mm, carotid = 1.5mm, and coronary = 1mm. Each vessel type was printed in 3 different material options. Material options are user-selected from the J750 printer software graphical user interface as blood vessel wall anatomy elements in ‘compliant’, ‘slightly compliant’, and ‘rigid’ options. Three replicates of each vessel type were printed in each of the three selected material options, for a total of 27 models. The vessels were connected to a flow loop system where pressure was monitored via a pressure wire and cross-sectional area was measured with intravascular ultrasound (IVUS). Distensibility was calculated by comparing the % difference in cross-sectional area vs. pulse pressure to clinical literature values. Target clinical ranges for normal and diseased population distensibility are 10.3-44 % for the aorta, 5.1-10.1 % for carotid artery, and 0.5-6 % for coronary artery.

**Results:**

Aorta test vessels had the most clinically representative distensibility when printed in user-selected ‘compliant’ and ‘slightly compliant’ material. All aorta test vessels of ‘compliant’ material (*n* = 3) and 2 of 3 ‘slightly compliant’ vessels evaluated were within target range. Carotid vessels were most clinically represented in distensibility when printed in ‘compliant’ and ‘slightly compliant’ material. For carotid test vessels, 2 of 3 ‘compliant’ material samples and 1 of 3 ‘slightly compliant’ material samples were within target range. Coronary arteries were most clinically represented in distensibility when printed in ‘slightly compliant’ and ‘rigid’ material. For coronary test vessels, 1 of 3 ‘slightly compliant’ materials and 3 of 3 ‘rigid’ material samples fell within target range.

**Conclusions:**

This study suggests that advancements in materials and 3D printing technology introduced with the J750 Digital Anatomy 3D Printer can enable anatomical models with clinically relevant distensibility.

## Background

Cardiovascular disease is the number one cause of death globally, creating a demand for accelerated and well-informed endovascular device development [1]. There is an evolving need for accurate vascular models to support this rapid device development. Currently, there are various benchtop models including 2D rigid [2], 3D printed (3DP) rigid [3], 3DP compliant [4], silicone [5], and *ex-vivo*platforms [6]. Although *in-vivo*testing is an essential phase of device development, benchtop models offer more durable and cost effective solutions, include a quicker turnaround time for manufacture, and have a longer shelf life [7–9].

Specifically, material jetting 3D-printing capabilities are evolving within the realm of medicine to provide clinicians and engineers with life-like patient-specific models for medical device development, physician training, surgical demonstration, and strategic procedural planning. These 3DP models can replicate patient-based vessel geometry within 125-microns [10]. In addition, material jetted 3D printed models can mimic disease states such as calcifications or lesions and be printed in various colors and material stiffnesses [11, 12]. To aid in simulating biomechanical properties of vessel walls, the vasculature may be printed using a variety of compliant materials. Material jetted 3D-printed anatomical models can be designed for use in a flow loop with physiological pressurized conditions for device testing under various imaging modalities such as planar x-ray [13], Computed Tomography [14], Magnetic Resonance [15] and ultrasound [16].

In the material jetting 3D printing community, there have been continuous efforts to replicate physiological characteristics in the 3D printed *in-vitro* models such as material selection, vessel geometry, lubricity, and elastic properties. Tabaczynski et al. characterized material properties of vascular models that best mimic healthy and diseased vessels. In addition, Tabaczynski’s other work has included characterizing vessel lubricity along a multi-material vessel path comprised of rigid reinforcements and compliant vessel wall which results in smooth and accurate device trackability within the vessel lumen [9, 11]. Furthermore, testing of different vessel wall thicknesses to vary vessel distensibility under a pressurized flow loop allows a more accurately represented *in-vitro*benchtop system [9, 11]. Studies conducted using a J750 3D Printer, prior to the release of the J750 Digital Anatomy 3D Printer, indicated that preset materials may not be sufficient to replicate the arterial wall with physiological accuracy [9, 11]. The J750 printer can employ multiple materials within a single printed model, which provides many options to vary mechanical properties. Stratasys’ J750 Digital Anatomy 3D Printer materials established six new preset blood vessel wall compliance options, thereby improving the ability to create vascular models with various biomechanical properties.

Arterial distensibility is defined as an artery’s capacity to expand in response to an increase in blood pressure [17]. Cross-sectional distensibility is calculated by the relative change in lumen area for a given pressure change (ΔA/A × ΔP, where ΔA is change in lumen cross-sectional area between systole and diastole, A is lumen cross-sectional area in diastole, and ΔP is local pulse pressure), expressed in units of %ΔA/100mmHg. Clinical values for distensibility are 10.3-44 % for aorta arterial vessels, 5.1-10.1 % for carotid arterial vessels, and 0.5-6 % for coronary arterial vessels [17–19]. To enhance the simulation of evaluating device performance by a clinician, vascular models that feature distensibility can be a useful biomechanical attribute. To mimic native distensibility, 3D-printed vascular models’ materials must functionally replicate vessel change in cross-sectional area under physiological blood pressures [20]. With the option of using J750 materials, there is a possibility to simulate clinically relevant distensibility in 3DP arterial vessels. The opportunity to select vessel compliance material on the printer’s software graphic user interface (GUI) could allow for simulation of a wide range of vessel distensibility and therefore a more accurate assessment of vascular devices.

Clinically, physicians routinely use intravascular ultrasound (IVUS) to characterize vasculature such as inner lumen geometry and cross-sectional area [18, 21]. The IVUS system outputs a cross sectional image which can be measured, and along with measuring pulse pressure, distensibility can be calculated. IVUS catheters detect inner lumen diameter by propagating sound waves to the blood vessel and computing the signal reflected from the walls. Although designed for use in native vasculature, 3D-printed material in a water flow loop provides a compatible *in-vitro* environment for performing measurements for this study. Using the same clinical IVUS equipment to measure 3D-printed vessels reduces error of converting data from other measurement systems, providing a direct comparison to clinically reported values.

In this study, we aim to evaluate the distensibility of various 3D printed vessels using a J750 printer and compare with distensibility values of *in-vivo* vessels reported in literature.

## Methods

### 3D printed vessel samples

Three different modeled artery types including aorta, carotid, and coronary were evaluated for cross-sectional distensibility using different material blends to understand changes across varying inner lumen diameters. Table 1identifies the vessel configuration study parameters produced on a material jetting printer (J750, Stratasys, Rehovot, Israel) [11]. The representative inner lumen diameter for each vessel type was selected from literature [22–25]. The vessel model was designed in Solidworks (Waltham, MA, USA) using a series of geometric sketches and extrusions. Wall thickness was selected based on previous experience for withstanding physiological pressure and demonstrating desired vessel behavior (Table 1) [11, 22, 23, 26, 27]. Vessels were designed as straight tubes to promote consistent cross-sectional measures when using the IVUS system. Vessels feature rigid adaptors on each end to interface with a flow loop fixture. The model was then converted to a stereolithography (STL) file and imported into Stratasys printing software (GrabCAD Print, version 1.36) for building print trays and material selection.

**Table 1:** Summary of study parameters [11, 22, 23, 26, 27]

Artery Type	Inner Diameter, ID [mm]	Wall Thickness, WT [mm]	Vessel Length, [mm]	Compliance Material Options	Samples
Aorta	30.0	3.0	150	Compliant	3
				Slightly Compliant	3
				Rigid	3
Carotid	7.0	1.5	150	Compliant	3
				Slightly Compliant	3
				Rigid	3
Coronary	3.0	1.0	115	Compliant	3
				Slightly Compliant	3
				Rigid	3

**Fig. 1:**
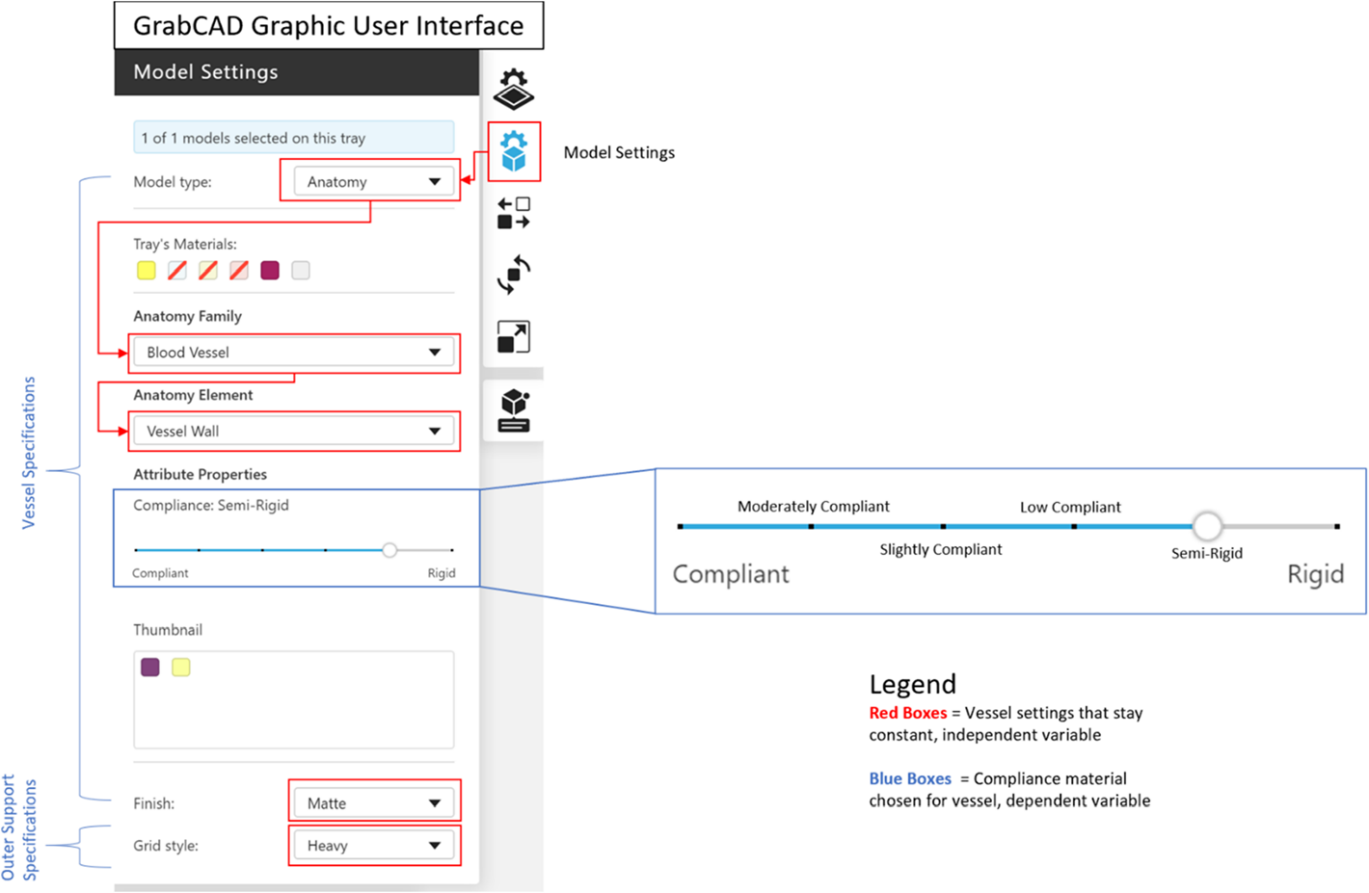
J750 Graphic User Interface (GUI), GrabCAD Print – Model Settings. On the GUI, the user can choose model type, view the materials that are in the printer at time of use, and select the desired anatomy type (anatomy family/element). These settings were the independent variable of the study in terms of vessel material selection and can be seen in the corresponding red boxes. The ‘attribute properties’ are the dependent variable in terms of vessel material selection and can be chosen along the sliding dial as seen in the corresponding blue boxes

GrabCAD Print offers six material combinations for blood vessel wall materials. The vessel wall materials are comprised of a mixture of a flexible material (Agilus30, shore hardness 30 A) and a rigid material (Vero, shore hardness 83D). The material mixture options in the software GUI provides 6 compliance options from ‘Compliant’ to ‘Rigid’ (Fig. 1) on a sliding dial. As the user slides from one compliance to another, the software algorithm changes the bulk material properties by affecting the ratio of Agilus30 to Vero.

**Fig. 2:**
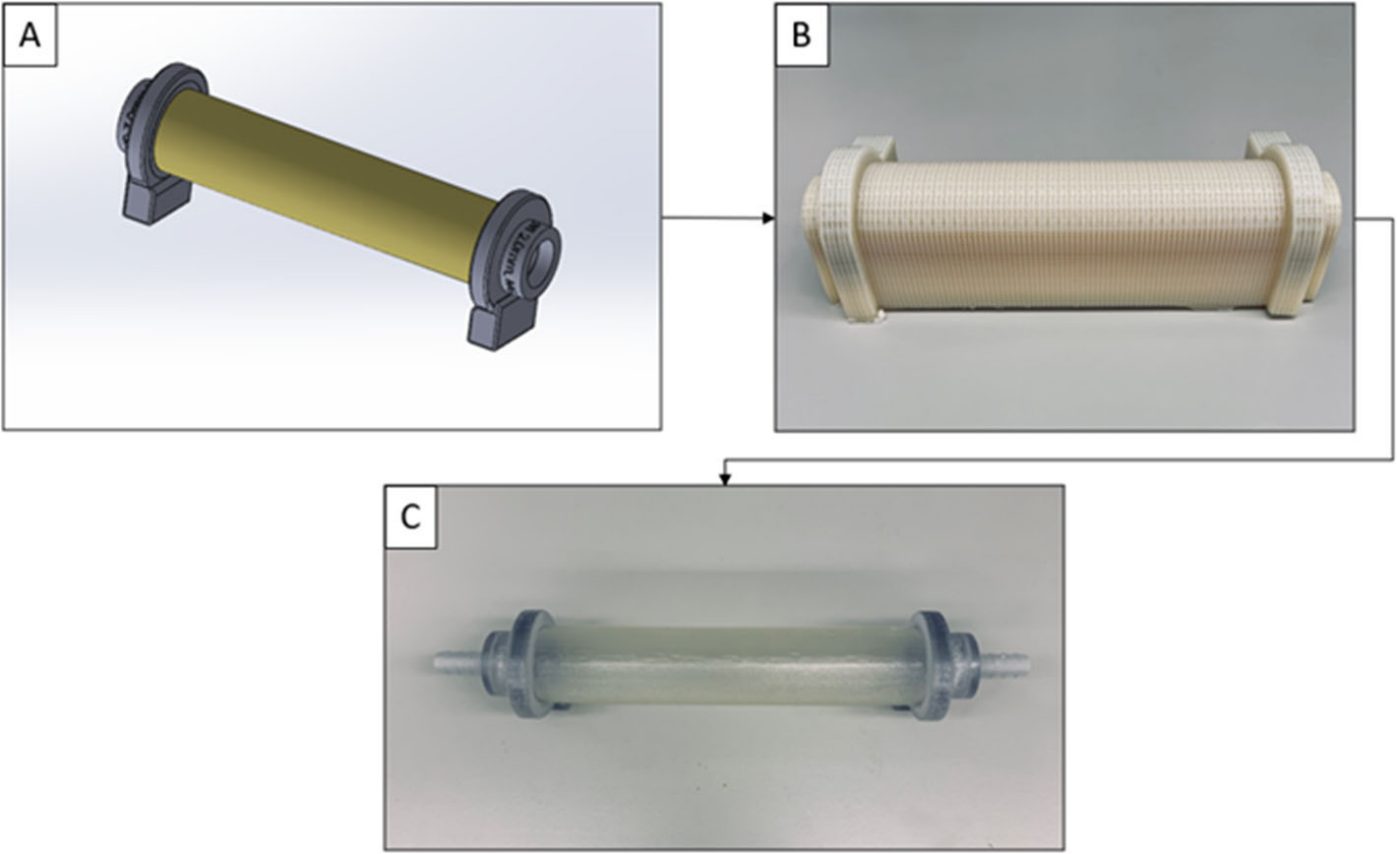
Vessel Model Creation Process. **A** Vessel with inlet and outlet supports designed and assembled in Solidworks, ready to print.; **B** Model successfully printed, still in support, ready for post-processing; **C** Model ready for testing with inlet and outlet connectors attached

The compliance option chosen for each vessel type for this study was ‘Compliant’, ‘Slightly Compliant’, and ‘Rigid’. Vessels were printed with GelSupport as internal support material with matte finish and heavy grid style as the outside support. Once successfully printed (Fig. 2B), support material was removed using standard model processing techniques. Water was used to rinse bulk support material from the models. An agitated bath of sodium hydroxide (2 % NaOH and 1 % Na_2_SiO_3_) was used to chemically clean models for 20 min. Once chemically cleaned, the models were rinsed with water and allowed to dry. Inlet and outlet connectors were attached to the rigid adaptors with adhesive (Fig. 2C). Post processing work per sample took approximately 30 min of hands-on labor.

### Flow system setup

**Fig. 3:**
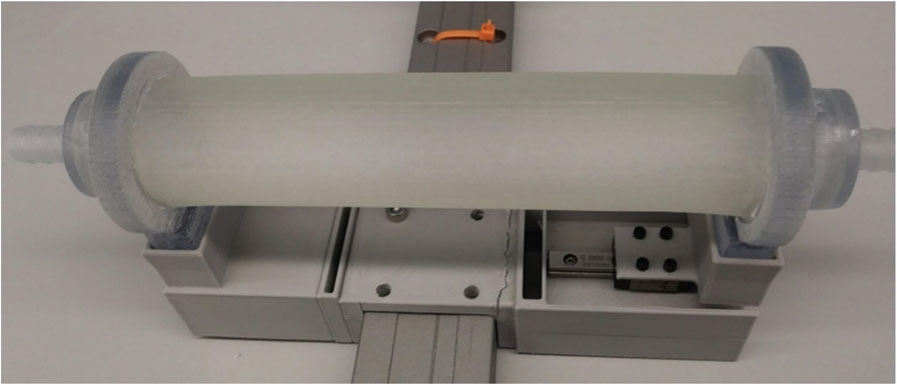
Test vessel fixture allowing axial movement: left side of vessel is fixed, right side of vessel is attached to a linear bearing

**Fig. 4:**
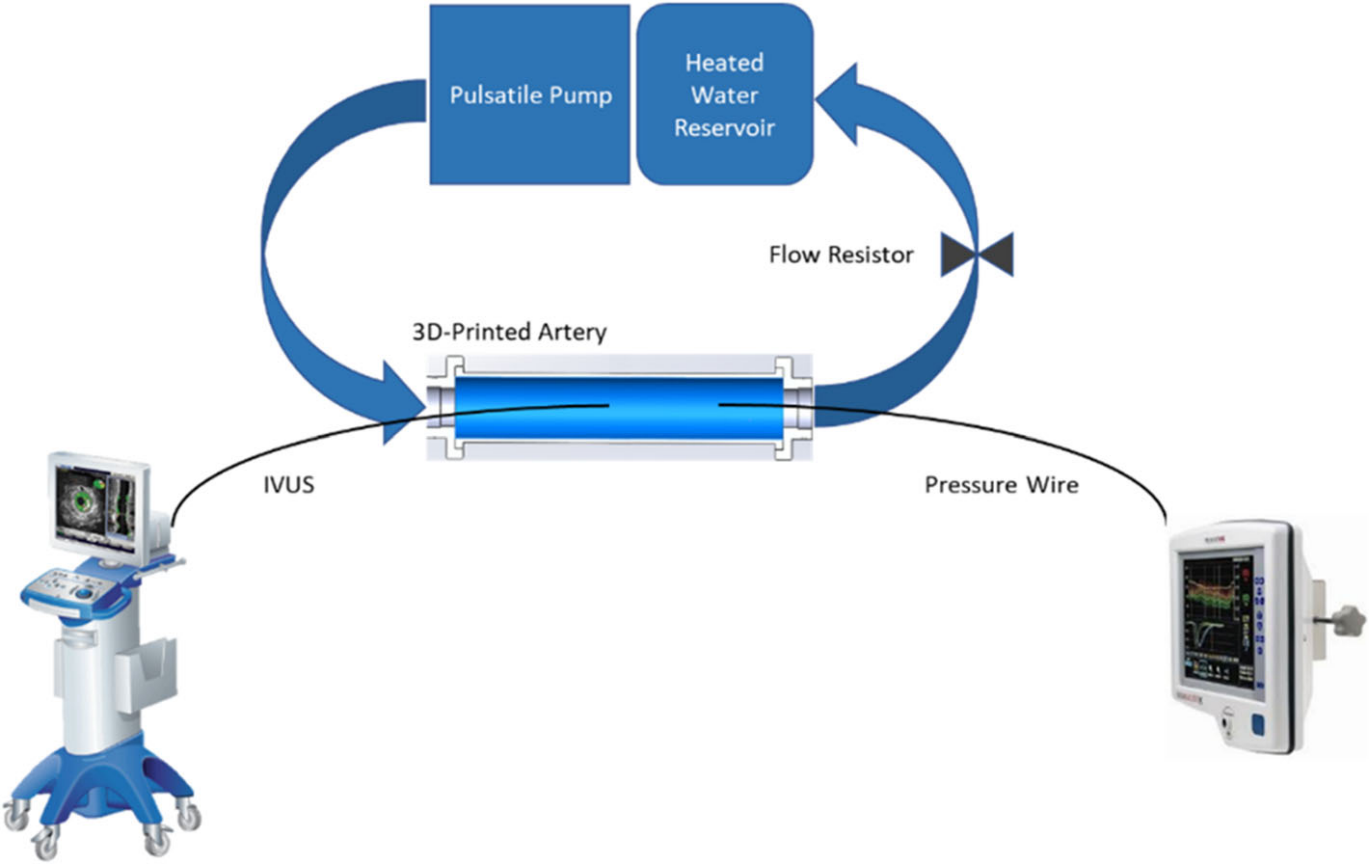
Flow System Setup

Each sample was installed onto a holding fixture which allowed the vessel ends to move freely in the axial direction. One side was anchored, while the other was attached to a linear bearing to allow motion along the length of the vessel (Fig. 3). Allowing the vessels to stretch along the length is important, as restricting the axial expansion could artificially increase the change in lumen area. The flow loop setup (Fig. 4) was consistent for all vascular models and testing was performed using a pulsatile pump (55-3305, Harvard Apparatus, MA, USA). The pulsatile pump provides cyclical pulsatile flow with an adjustable cardiac output and is connected to the flow model’s inlet via silicone tubing. Silicone tubing is connected to the outlet and to the heated reservoir system. A flow restrictor was used on the outflow tubing to help achieve target pressures. Water was circulated through the flow loop system and water bath temperature was maintained at 37.5 ± 0.5 °C. The target temperature of 37.5 °C was selected to mimic human body temperature, and temperature conditions were controlled as material properties can change with temperature. Target pressure of 120/80mmHg was selected to mimic physiological values, with a mean arterial pressure (MAP) of 100 ± 5mmHg and pulse pressure of 40 ± 5mmHg [24, 25, 28]. MAP and pulse pressure were monitored via a Volcano pressure wire system (Andover, MA, USA) and regulated per vessel type by adjusting the pump volume/stroke setting. Pulsatile pump diastolic/systolic phase ratio and rate remained constant at 35/65 % phase ratio, and 60 revolutions per minute, respectively [29, 30]. Air bubbles were eliminated via an in-line compliance chamber included in the inlet tubing. Volume of water in the compliance chamber was adjusted in conjunction with pump volume/stroke output until target pressure conditions were met.

### Distensibility testing

#### IVUS image measurements

An IVUS catheter specified for the corresponding inner lumen diameter was positioned at center-length within each vessel. Philips (Andover, MA, USA) IVUS catheters (Eagle Eye Platinum ST or Visions PV8.2) were cross-sectionally centered with rail-assistance via an .035” Terumo (Somerset, NJ, USA) guide wire (Glidewire) tracked through the entire sample. Once clinically relevant hemodynamics were established, conditions were held for 2 min before measurements began, with stability confirmed by unchanged MAP value between 0 and 2 min. A 15 s IVUS reading was recorded. Pressure (MAP, Systolic, Diastolic, and Pulse Pressure) was also simultaneously recorded during this time.

**Fig. 5:**
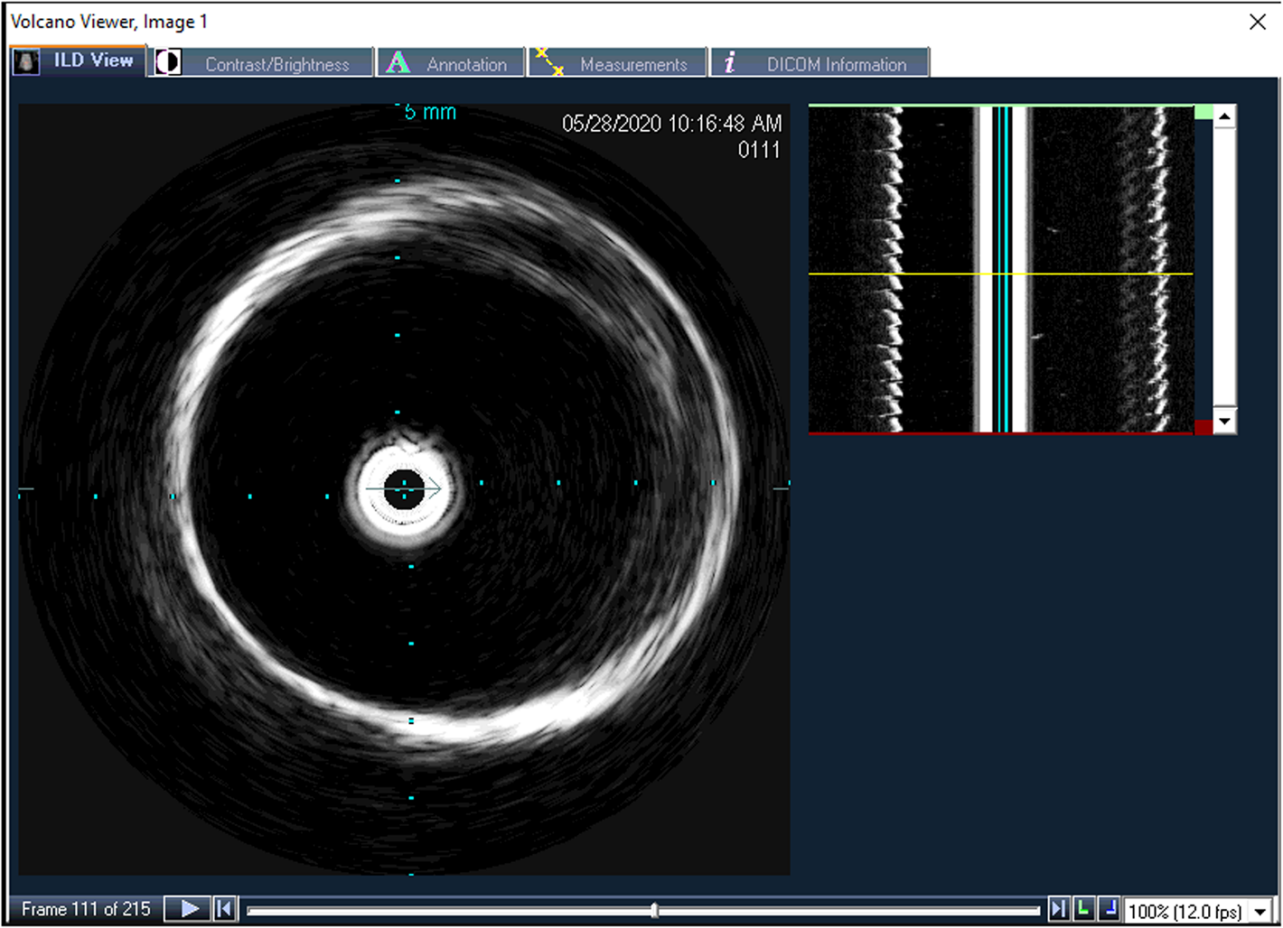
IVUS In-line Digital Display layout shows the transverse view of an artery on the left and the corresponding time-lapse longitudinal view on the right. The yellow bar in the longitudinal view is a cursor control defining the time of the transverse view, with time increasing as the yellow bar moves down. The arrow in the center of the transverse view is a cursor control for defining the angular orientation of the longitudinal view. The diastole and systole positions are identified by the widest and narrowest points within the longitudinal view

The IVUS hardware captures videos of the pulsating vessels at 12 to 30 frames per second. Using the longitudinal view, the user visually identified locations of minimum diameter, representing diastole and maximum diameter representing systole for three separate cardiac cycles (Fig. 5). Cross-sectional area (mm^2^) was then measured from each image using the IVUS console software.

#### Pressure wire measurements

**Fig. 6:**
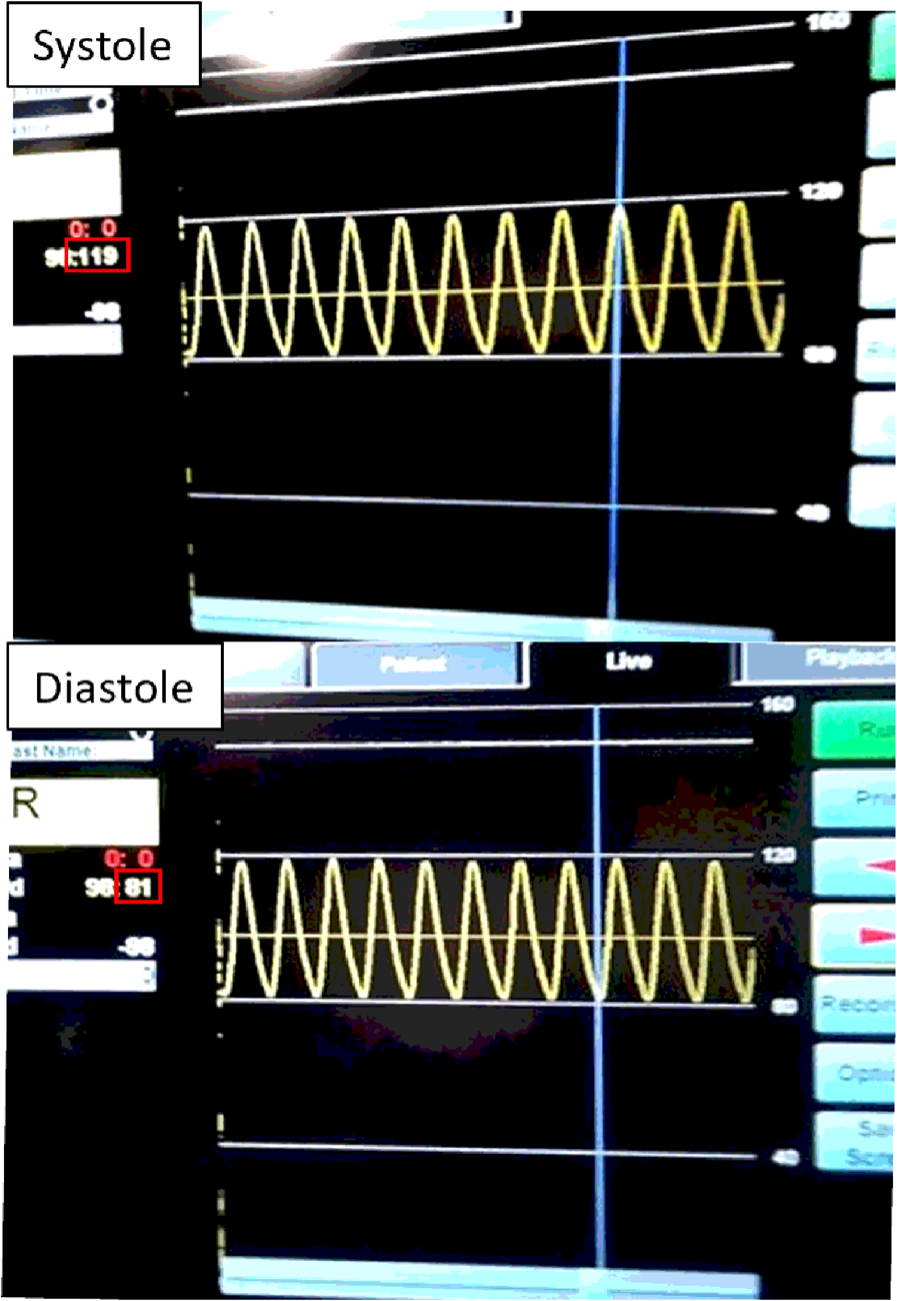
Pressure wire display layout shows the pressure value over time. The vertical line is a cursor finding the time of the pressure data point

Pressure was captured continuously from the Volcano system at 60 Hz. The minimum, maximum, and MAP were obtained from the captured pressure waveform (Fig. 6).

### Target values & distensibility determination

Distensibility was calculated per equations below. Units are expressed as %ΔArea / 100mmHg:

1 $$\text{D}\text{i}\text{s}\text{t}\text{e}\text{n}\text{s}\text{i}\text{b}\text{i}\text{l}\text{i}\text{t}\text{y} \left[\frac{\text{\%}{\Delta }\text{A}\text{r}\text{e}\text{a}}{100\text{m}\text{m}\text{H}\text{g}}\right] =\frac{ \frac{\text{M}\text{a}\text{x}\text{i}\text{m}\text{u}\text{m} \text{L}\text{u}\text{m}\text{e}\text{n} \text{A}\text{r}\text{e}\text{a} \left[{\text{m}\text{m}}^{2}\right]-\text{M}\text{i}\text{n}\text{i}\text{m}\text{u}\text{m} \text{L}\text{u}\text{m}\text{e}\text{n} \text{A}\text{r}\text{e}\text{a} \left[{\text{m}\text{m}}^{2}\right]}{\text{M}\text{i}\text{n}\text{i}\text{m}\text{u}\text{m} \text{L}\text{u}\text{m}\text{e}\text{n} \text{A}\text{r}\text{e}\text{a} \left[{\text{m}\text{m}}^{2}\right]}}{\text{P}\text{u}\text{l}\text{s}\text{e} \text{P}\text{r}\text{e}\text{s}\text{s}\text{u}\text{r}\text{e} \left[\text{m}\text{m}\text{H}\text{g}\right]\times 100}$$

2 $$\text{P}\text{u}\text{l}\text{s}\text{e} \text{P}\text{r}\text{e}\text{s}\text{s}\text{u}\text{r}\text{e} \left[\text{m}\text{m}\text{H}\text{g}\right]= \text{S}\text{y}\text{s}\text{t}\text{o}\text{l}\text{i}\text{c} \left(\text{M}\text{a}\text{x}\text{i}\text{m}\text{u}\text{m}\right)\text{P}\text{r}\text{e}\text{s}\text{s}\text{u}\text{r}\text{e} \left[\text{m}\text{m}\text{H}\text{g}\right] - \text{D}\text{i}\text{a}\text{s}\text{t}\text{o}\text{l}\text{i}\text{c} \left(\text{M}\text{i}\text{n}\text{i}\text{m}\text{u}\text{m}\right)\text{P}\text{r}\text{e}\text{s}\text{s}\text{u}\text{r}\text{e}\left[\text{m}\text{m}\text{H}\text{g}\right]$$

Distensibility results for each sample were then compared to the corresponding target clinical ranges reported in available literature (Table 2) [17–19].

**Table 2:** Clinical target distensibility per artery type

Artery Type	Target Distensibility [ % ΔArea / 100mmHg]	Population
Aorta	10.3–44.0 [18]	57 ± 11 year (standard deviation), normal & diseased
Carotid	5.1–10.1 [27]	21–75 year, normal & hypertensive
Coronary	0.5–6.0 [26]	50–85 year, healthy & diseased

## Results

### Testing conditions

All vessel test conditions achieved target MAP and pulse pressure. Table 3, below, identifies mean recorded MAP and pulse pressure for each tested vessel type.

**Table 3:** Hemodynamics across all material types during testing

Artery Type	Mean MAP [mmHg]	Mean Pulse Pressure [mmHg]
Aorta (*n* = 9)	99.6 ± 0.7	40.1 ± 0.7
Carotid (*n* = 9)	99.2 ± 0.9	40.2 ± 1.5
Coronary (*n* = 9)	98.6 ± 1.9	40.4 ± 1.8

Results reported as Mean ± Standard Deviation

**Fig. 7:**
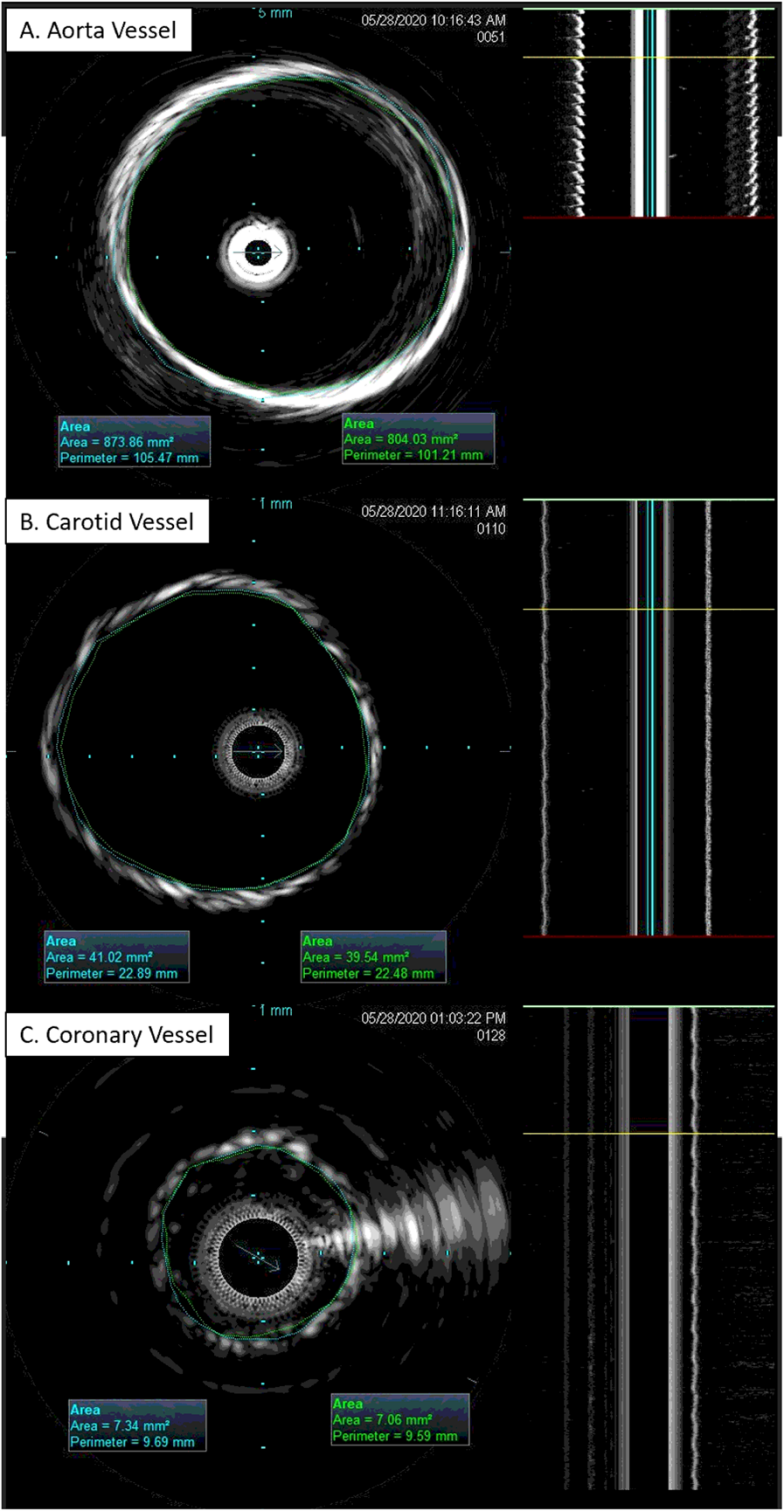
Sample IVUS cross-sectional area measurements of systolic (blue) and diastolic (green) conditions. Frames **A-C** are in the diastolic position of each vessel. Sample vessels **A-C** were printed in the ‘compliant’ material

All vessel cross-sectional area measurements were successfully taken using the IVUS software (Fig. 7). The area measurements were then used for distensibility calculations.

### Distensibility

**Fig. 8:**
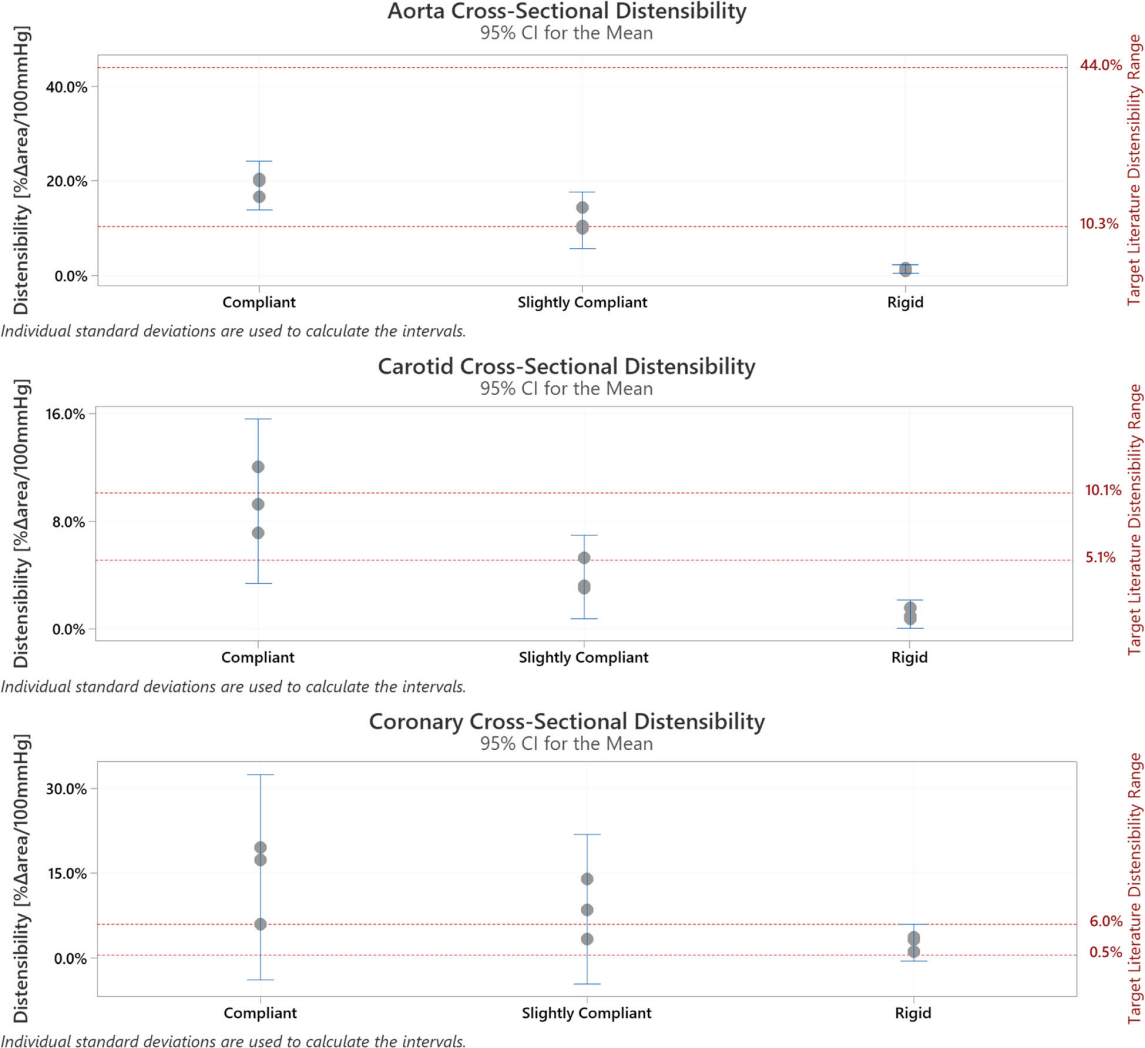
Distensibility Results

Nine samples of each vessel type were tested. There were three replicates of each of the three materials: ‘compliant’, ‘semi compliant’, and ‘rigid’. Compliant exhibited the highest distensibility, whereas rigid corresponded with the lowest distensibility. The measured distensibility values had a decreasing trend as materials moved from compliant to rigid (Fig. 8).

#### Aorta cross-sectional distensibility

Figure 8 (top graph) is an individual value plot displaying calculated distensibility results per material type where target literature range is represented by the area between the 2 dotted red lines. ‘Compliant’ (3 of 3 samples) and ‘slightly compliant’ (2 of 3 samples) fell within the target range for aorta cross sectional distensibility.

#### Carotid cross-sectional distensibility

An individual value plot displaying calculated distensibility results per material type where target literature range is represented by the area between the two dotted red lines (Fig. 8, middle graph). ‘Compliant’ (2 of 3 samples) and ‘slightly compliant’ (1 of 3 samples) vessels fell within the target range for carotid cross sectional distensibility.

#### Coronary cross-sectional distensibility

Figure 8 (bottom graph) consists of an individual value plot displaying calculated distensibility results per material type where target literature range is represented by the area between the 2 dotted red lines. ‘Slightly compliant’ (1 of 3 samples) and ‘rigid’ (3 of 3 samples) vessels fell within the target range for coronary cross sectional distensibility.

Based on the results of our study, recommended material assignment and wall thickness have been identified for the vessel types analyzed (Table 4).

**Table 4:** Recommended design parameters for distensible arteries

Vessel Type	Inner Diameter [mm]	Wall Thickness [mm]	Material
**Aorta**	30	3	Compliant, Slightly Compliant
**Carotid**	9	1.5	Compliant, Slightly Compliant
**Coronary**	3	1	Slightly Compliant, Rigid

## Discussion

Simulation of endovascular intervention demands clinical relevancy, and material jetting 3D printing presents a solution for mimicking vascular distensibility. For the first time, 3D printed vascular models on a J750 were analyzed using intravascular ultrasound and successfully demonstrated the potential to accurately represent the distensibility of human arteries.

Target distensibility values were referenced from clinical studies varying in patient disease-state, sex, and age range. The use of IVUS allows for the capture of dynamic response, which is the only way to capture the non-linear response of the material in pulsatile physiological conditions. While static measurements may be more stable, it introduces error related to how the material responds dynamically due to its elastic properties. A vessel at static pressure may not have the same cross-sectional area as a vessel in physiological pulsatile flow at that same pressure. The clinical data was captured dynamically, therefore the test conditions were matched to make the most direct comparison.

By integrating controlled mechanical properties to vascular anatomical models, the utility of these models can be expanded to perform meaningful endovascular device testing in known physiological conditions. Recognizing that both material and geometrical factors contribute to the overall model properties, there is significant opportunity to improve the clinical accuracy of these models. In the endovascular space, devices traverse through the blood vessels to reach target locations and perform specific functions. The distensibility of a blood vessel is a key factor in endovascular device performance metrics such as the ability to track a device to a desired location, how a stent maintains its position within an artery when deployed, or how a device fills an aneurysm.

This feasibility study is limited by a small sample size, simplified anatomical test geometries, and limited blood vessel locations. There are other features important to vascular models that were not explored. The focus of this study is distensibility, but other properties such as lubricity, clarity, and toughness are also important features for vascular models.

Further characterization testing could increase the understanding of the relationships of wall thickness and vessel diameter to distensibility, which could inform the creation of a look-up chart to select parameters to reach a target distensibility. Further research into material modulation, including voxel-based or multi-layer designs, will contribute to the ability to design arteries representing more specific populations i.e., healthy vs. diseased, old vs. young.

The results of this study indicate the J750 provides an appropriate range of arterial distensibility to fit research and clinical needs in 3D printed vascular models.

## Conclusions

When simulating arteries for treatment planning, education, and product testing, the distensibility of arteries is important in understanding how the artery will move as internal and external forces are applied. Arteries are dynamic structures that expand as a result of internal blood pressures. This study suggests the J750 and its associated materials can create arterial models that are biomechanically at, or close to, target physiological values representing a generalized population of healthy and diseased vessels. The realism of models produced by the J750 can provide tremendous value by enabling use of models with accurate distensibility in simulations. In addition, this study demonstrates that it is both feasible and appropriate to utilize IVUS as an appropriate method to characterize distensibility in vascular models.

## Data Availability

The data that support the findings of this study are available from The Jacobs Institute, but restrictions apply to the availability of these data, which were used under license for the current study, and so are not publicly available. Data are however available from the authors upon reasonable request and with permission of The Jacobs Institute.

## Abbreviations

3DP: 3D Printing
GUI: Graphic User Interface
IVUS: Intravascular Ultrasound
MAP: Mean Arterial Pressure
STL: STereoLithography
